# Supplementary material for: Integrated Metabolite and Transcriptome Profiling-Mediated Gene Mining of Sida cordifolia Reveals Medicinally Important Genes
Source: Genes (Basel). 2022 Oct 20;13(10):1909. doi: 10.3390/genes13101909 (PMC9602365; doi:10.3390/genes13101909)
Supplement: Supplementary file 1 [file genes-13-01909-s001.zip › LCMS of Leaf.pdf]

NAWAL ANALYTICAL LABORATORIES HOSUR

Sample Information

Sample Name	: SL
Sample ID	: SL
Tray#	: 2
Vial#	: 52
Injection Volume	: 20
Data File	: SL.lcd

MS Chromatogram

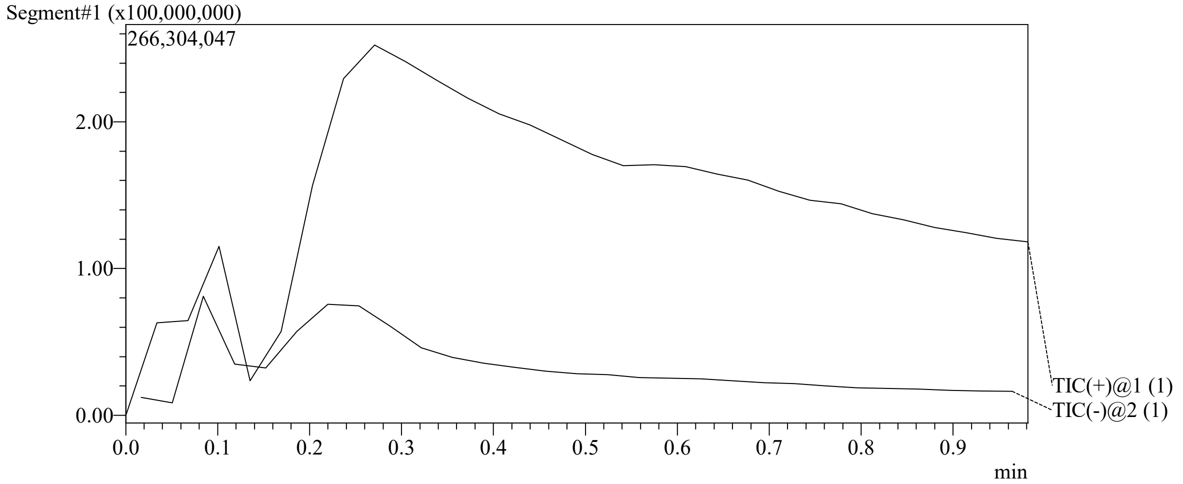

MS Spectrum
SL.lcd

Line#:1 R.Time:----(Scan#:----)
MassPeaks:29
Spectrum Mode:Averaged 0.000-0.846(1-51) Base Peak:306(67634)
BG Mode:Averaged 0.000-0.880(1-53) Segment 1 - Event 1

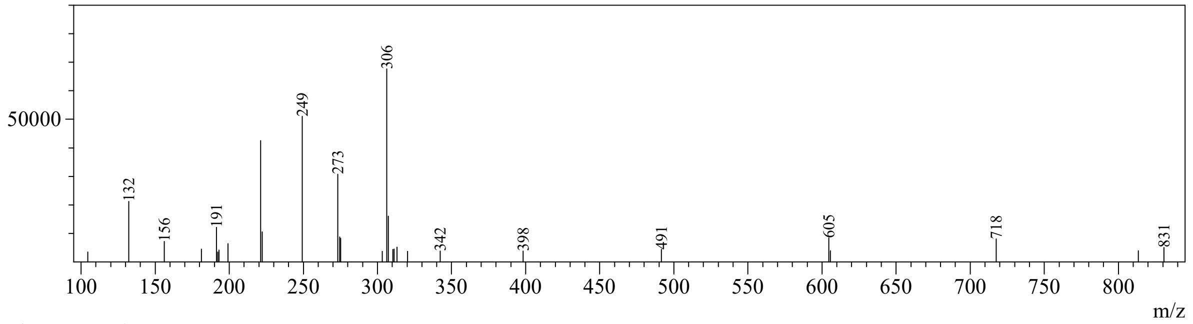

Line#:2 R.Time:----(Scan#:----)
MassPeaks:89
Spectrum Mode:Averaged 0.017-0.863(2-52) Base Peak:714(22756)
BG Mode:Averaged 0.017-0.897(2-54) Segment 1 - Event 2

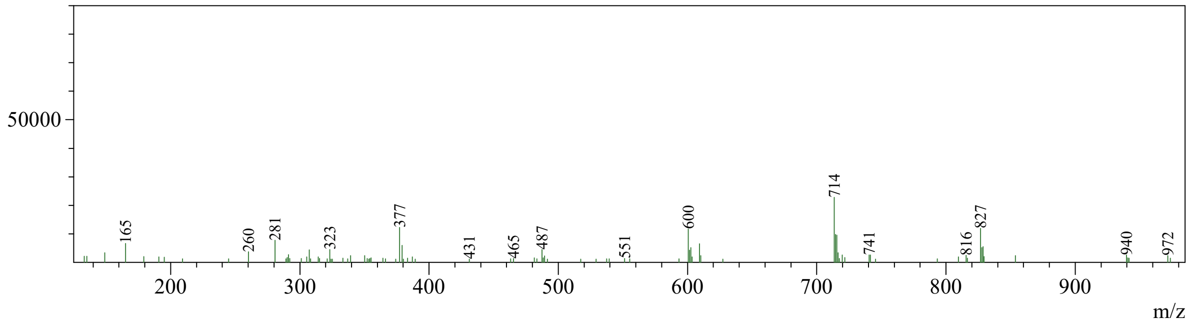

NAWAL ANALYTICAL LABORATORIES HOSUR

Line#:3 R.Time:----(Scan#:----)
MassPeaks:29
Spectrum Mode:Averaged 0.000-0.846(1-51) Base Peak:306(67634)
BG Mode:Averaged 0.000-0.880(1-53) Segment 1 - Event 1

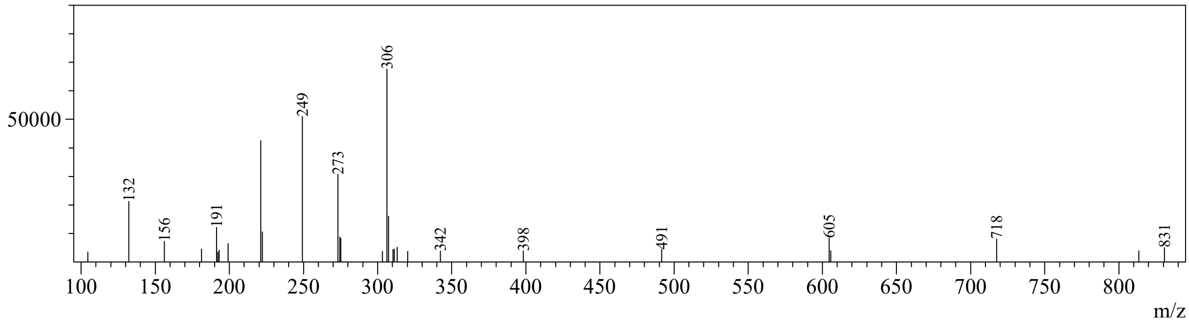

Line#:4 R.Time:----(Scan#:----)
MassPeaks:89
Spectrum Mode:Averaged 0.017-0.863(2-52) Base Peak:714(22756)
BG Mode:Averaged 0.017-0.897(2-54) Segment 1 - Event 2

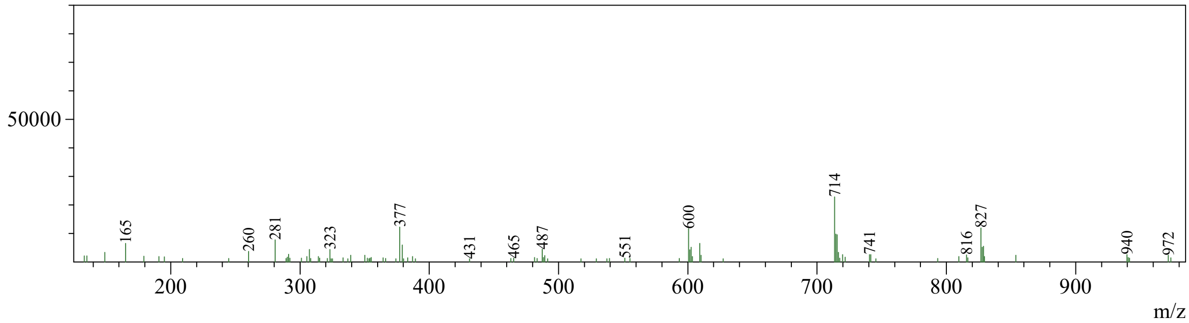

MS Spectrum

Line#:1 R.Time:----(Scan#:----)
MassPeaks:29
Spectrum Mode:Averaged 0.000-0.846(1-51) Base Peak:306(67634)
BG Mode:Averaged 0.000-0.880(1-53) Segment 1 - Event 1

#	m/z	Absolute Intensity	Relative Intensity
1	104.50	3433	5.08
2	132.15	21239	31.40
3	156.20	7147	10.57
4	181.15	4473	6.61
5	191.25	12097	17.89
6	192.20	3527	5.21
7	193.15	4120	6.09
8	199.15	6359	9.40
9	221.20	42531	62.88
10	222.25	10574	15.63
11	249.20	51078	75.52
12	273.15	30749	45.46
13	274.40	8796	13.01
14	275.20	8254	12.20
15	303.25	3662	5.41
16	306.25	67634	100.00
17	307.25	16038	23.71
18	310.40	4355	6.44
19	311.20	4434	6.56
20	313.20	5143	7.60
21	320.30	3734	5.52

NAWAL ANALYTICAL LABORATORIES HOSUR

#	m/z	Absolute Intensity	Relative Intensity
22	342.40	3835	5.67
23	398.25	3869	5.72
24	491.55	4389	6.49
25	604.60	8470	12.52
26	605.65	3938	5.82
27	717.65	8124	12.01
28	813.40	3964	5.86
29	830.70	5052	7.47

Line#:2 R.Time:----(Scan#:----)
MassPeaks:89
Spectrum Mode:Averaged 0.017-0.863(2-52) Base Peak:714(22756)
BG Mode:Averaged 0.017-0.897(2-54) Segment 1 - Event 2

#	m/z	Absolute Intensity	Relative Intensity
1	133.25	2162	9.50
2	135.20	2167	9.52
3	149.15	3393	14.91
4	165.10	6569	28.87
5	179.20	1974	8.67
6	191.00	1962	8.62
7	195.10	1823	8.01
8	209.15	1281	5.63
9	245.00	1257	5.52
10	260.20	3719	16.34
11	280.90	7751	34.06
12	289.10	1198	5.26
13	290.20	1667	7.33
14	291.25	2709	11.90
15	292.15	1328	5.84
16	301.20	1295	5.69
17	305.20	1890	8.31
18	307.25	4347	19.10
19	308.20	1185	5.21
20	314.25	1868	8.21
21	315.15	1315	5.78
22	321.25	1251	5.50
23	323.20	4435	19.49
24	324.25	1141	5.01
25	325.15	1151	5.06
26	333.20	1505	6.61
27	337.10	1179	5.18
28	339.30	2311	10.16
29	350.20	2325	10.22
30	352.20	1361	5.98
31	353.30	1153	5.07
32	354.15	1360	5.98
33	355.20	1567	6.89
34	364.35	1415	6.22
35	366.20	1212	5.33
36	374.20	1175	5.16

NAWAL ANALYTICAL LABORATORIES HOSUR

#	m/z	Absolute Intensity	Relative Intensity
37	377.20	12251	53.84
38	379.15	5954	26.16
39	380.05	1147	5.04
40	383.30	1422	6.25
41	387.15	1890	8.31
42	389.25	1155	5.08
43	431.25	1155	5.08
44	463.20	1260	5.54
45	465.30	1302	5.72
46	481.35	1598	7.02
47	483.40	1289	5.66
48	487.45	4440	19.51
49	488.40	1564	6.87
50	489.45	2213	9.72
51	491.50	1165	5.12
52	517.30	1174	5.16
53	529.30	1165	5.12
54	537.35	1179	5.18
55	539.35	1186	5.21
56	551.35	1295	5.69
57	555.30	1195	5.25
58	593.30	1194	5.25
59	600.50	11636	51.13
60	601.50	4296	18.88
61	602.50	5201	22.86
62	603.50	1944	8.54
63	609.25	6525	28.67
64	610.30	2409	10.59
65	627.45	1145	5.03
66	713.60	22756	100.00
67	714.60	9815	43.13
68	715.60	9496	41.73
69	716.55	3327	14.62
70	717.40	1364	5.99
71	719.60	2580	11.34
72	721.70	1687	7.41
73	740.60	2620	11.51
74	741.60	2539	11.16
75	745.55	1158	5.09
76	793.40	1188	5.22
77	809.60	1926	8.46
78	815.60	2503	11.00
79	816.65	1423	6.25
80	826.65	11921	52.39
81	827.65	5149	22.63
82	828.65	5517	24.24
83	829.55	1965	8.64
84	853.70	2369	10.41

#	m/z	Absolute Intensity	Relative Intensity
85	939.70	2573	11.31
86	940.70	1545	6.79
87	941.70	1404	6.17
88	971.60	2402	10.56
89	973.55	1471	6.46

Line#:3 R.Time:----(Scan#:----)
MassPeaks:29
Spectrum Mode:Averaged 0.000-0.846(1-51) Base Peak:306(67634)
BG Mode:Averaged 0.000-0.880(1-53) Segment 1 - Event 1

#	m/z	Absolute Intensity	Relative Intensity
1	104.50	3433	5.08
2	132.15	21239	31.40
3	156.20	7147	10.57
4	181.15	4473	6.61
5	191.25	12097	17.89
6	192.20	3527	5.21
7	193.15	4120	6.09
8	199.15	6359	9.40
9	221.20	42531	62.88
10	222.25	10574	15.63
11	249.20	51078	75.52
12	273.15	30749	45.46
13	274.40	8796	13.01
14	275.20	8254	12.20
