# Supplementary material for: Integrated Metabolite and Transcriptome Profiling-Mediated Gene Mining of Sida cordifolia Reveals Medicinally Important Genes
Source: Genes (Basel). 2022 Oct 20;13(10):1909. doi: 10.3390/genes13101909 (PMC9602365; doi:10.3390/genes13101909)
Supplement: Supplementary file 1 [file genes-13-01909-s001.zip › LCMS of root.pdf]

NAWAL ANALYTICAL LABORATORIES HOSUR

Sample Information

Sample Name	: SR
Sample ID	: SR
Tray#	: 2
Vial#	: 50
Injection Volume	: 20
Data File	: SR.lcd

MS Chromatogram

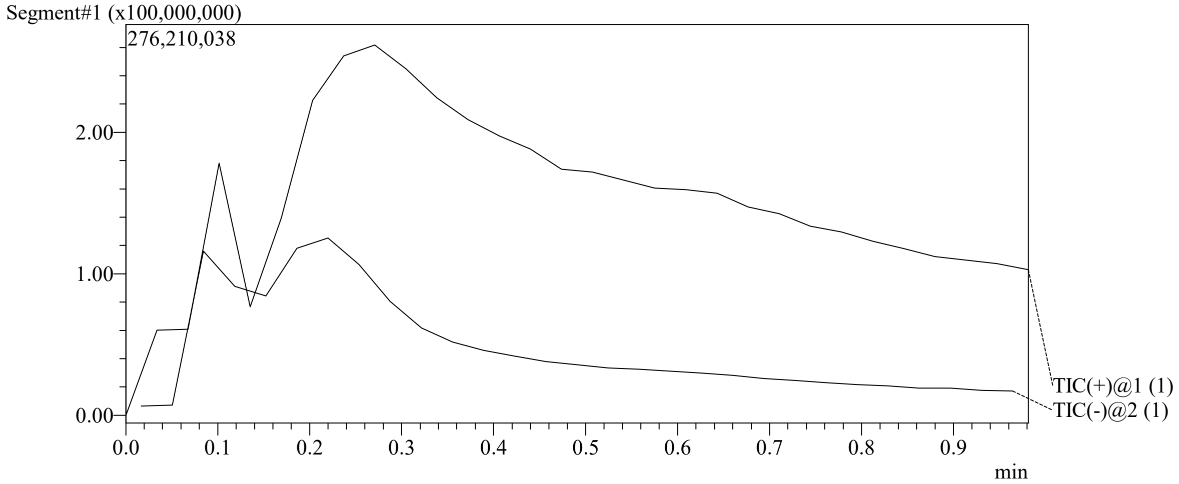

MS Spectrum
SR.lcd

Line#:1 R.Time:----(Scan#:----)
MassPeaks:22
Spectrum Mode:Averaged 0.000-0.914(1-55) Base Peak:118(8233057)
BG Mode:None Segment 1 - Event 1

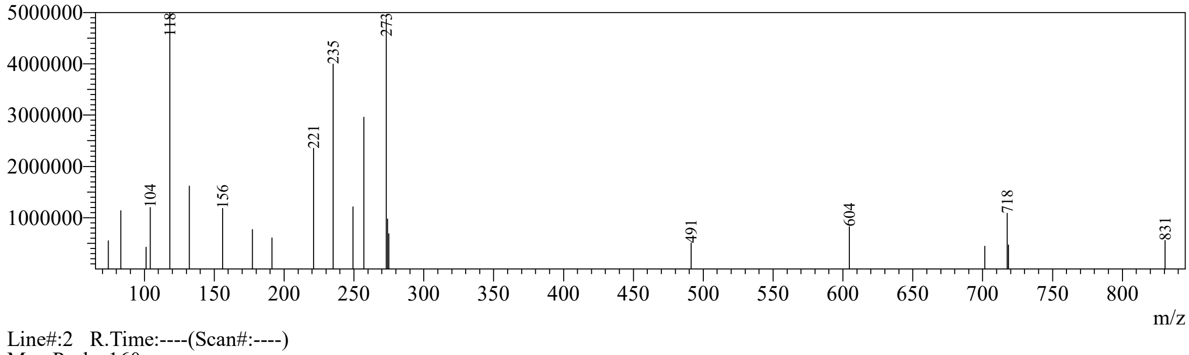

Line#:2 R.Time:----(Scan#:----)
MassPeaks:160
Spectrum Mode:Averaged 0.017-0.930(2-56) Base Peak:225(1065894)
BG Mode:None Segment 1 - Event 2

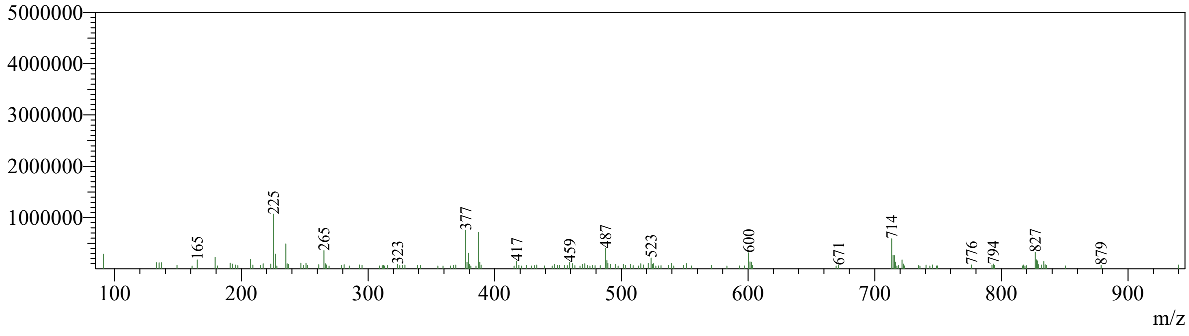

MS Spectrum

Line#:1 R.Time:----(Scan#:----)
MassPeaks:22
Spectrum Mode:Averaged 0.000-0.914(1-55) Base Peak:118(8233057)
BG Mode:None Segment 1 - Event 1

NAWAL ANALYTICAL LABORATORIES HOSUR

#	m/z	Absolute Intensity	Relative Intensity
1	74.10	552027	6.71
2	83.05	1132977	13.76
3	101.25	425138	5.16
4	104.25	1199500	14.57
5	118.20	8233057	100.00
6	132.15	1615978	19.63
7	155.95	1180758	14.34
8	177.20	770359	9.36
9	191.25	608286	7.39
10	221.20	2351699	28.56
11	235.15	3996560	48.54
12	249.20	1211596	14.72
13	257.15	2958133	35.93
14	273.10	4873579	59.20
15	274.15	971460	11.80
16	275.05	686713	8.34
17	491.35	507245	6.16
18	604.45	823060	10.00
19	701.55	441977	5.37
20	717.60	1083664	13.16
21	718.55	471117	5.72
22	830.65	553024	6.72

Line#:2 R.Time:----(Scan#:----)
MassPeaks:160
Spectrum Mode:Averaged 0.017-0.930(2-56) Base Peak:225(1065894)
BG Mode:None Segment 1 - Event 2

#	m/z	Absolute Intensity	Relative Intensity
1	91.30	287636	26.99
2	133.10	118949	11.16
3	135.10	117982	11.07
4	137.15	115776	10.86
5	149.15	71497	6.71
6	161.15	57566	5.40
7	165.15	173260	16.25
8	179.15	225585	21.16
9	181.15	56181	5.27
10	191.10	111341	10.45
11	193.10	91870	8.62
12	195.15	74362	6.98
13	197.15	59731	5.60
14	207.10	189965	17.82
15	209.15	72177	6.77
16	215.15	60485	5.67
17	217.15	97284	9.13
18	223.15	92085	8.64
19	225.15	1065894	100.00
20	227.15	286805	26.91
21	228.10	54372	5.10
22	235.15	487511	45.74

NAWAL ANALYTICAL LABORATORIES HOSUR

#	m/z	Absolute Intensity	Relative Intensity
23	236.15	98523	9.24
24	237.10	85037	7.98
25	247.10	110342	10.35
26	249.10	65527	6.15
27	251.15	115054	10.79
28	252.15	71465	6.70
29	261.10	79202	7.43
30	265.15	354783	33.29
31	266.20	99191	9.31
32	267.15	77765	7.30
33	269.20	55683	5.22
34	279.15	65687	6.16
35	281.15	82428	7.73
36	285.20	55604	5.22
37	293.20	77858	7.30
38	295.15	70462	6.61
39	309.20	57424	5.39
40	311.25	64137	6.02
41	312.20	61959	5.81
42	313.20	55752	5.23
43	315.20	61940	5.81
44	323.25	89975	8.44
45	325.20	64951	6.09
46	327.20	65595	6.15
47	329.20	71854	6.74
48	339.25	70302	6.60
49	341.15	68245	6.40
50	355.25	58810	5.52
51	359.20	58233	5.46
52	365.25	54028	5.07
53	367.20	66993	6.29
54	369.25	76209	7.15
55	377.20	755927	70.92
56	378.20	133273	12.50
57	379.20	303090	28.44
58	380.20	79593	7.47
59	381.25	58303	5.47
60	385.20	54461	5.11
61	387.20	710584	66.67
62	388.20	133731	12.55
63	389.20	75635	7.10
64	415.25	54714	5.13
65	417.20	151703	14.23
66	419.20	67092	6.29
67	421.25	54182	5.08
68	425.25	63231	5.93
69	429.25	56946	5.34
70	431.25	62870	5.90

NAWAL ANALYTICAL LABORATORIES HOSUR

#	m/z	Absolute Intensity	Relative Intensity
71	433.25	76497	7.18
72	439.25	59255	5.56
73	445.25	55718	5.23
74	447.25	83060	7.79
75	449.30	66189	6.21
76	451.25	67195	6.30
77	455.30	64215	6.02
78	457.25	61478	5.77
