# Supplementary material for: Integrated Metabolite and Transcriptome Profiling-Mediated Gene Mining of Sida cordifolia Reveals Medicinally Important Genes
Source: Genes (Basel). 2022 Oct 20;13(10):1909. doi: 10.3390/genes13101909 (PMC9602365; doi:10.3390/genes13101909)
Supplement: Supplementary file 1 [file genes-13-01909-s001.zip › LCMS of stem.pdf]

NAWAL ANALYTICAL LABORATORIES HOSUR

Sample Information

Sample Name	: SS
Sample ID	: SS
Tray#	: 2
Vial#	: 51
Injection Volume	: 20
Data File	: SS.lcd

MS Chromatogram

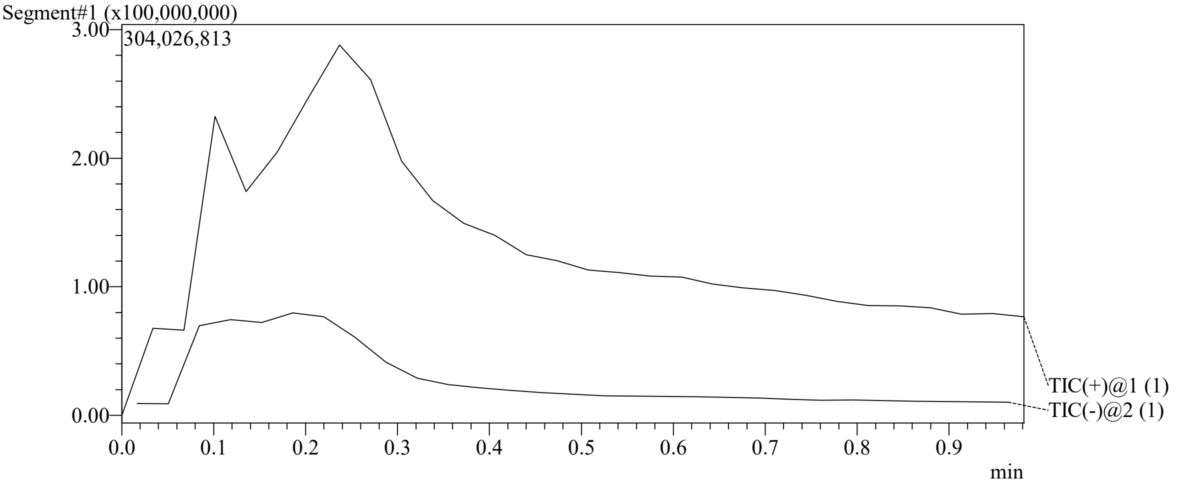

MS Spectrum

SS.lcd

Line#:1 R.Time:----(Scan#:----)
MassPeaks:36
Spectrum Mode:Averaged 0.000-0.914(1-55) Base Peak:118(6370435)
BG Mode:None Segment 1 - Event 1

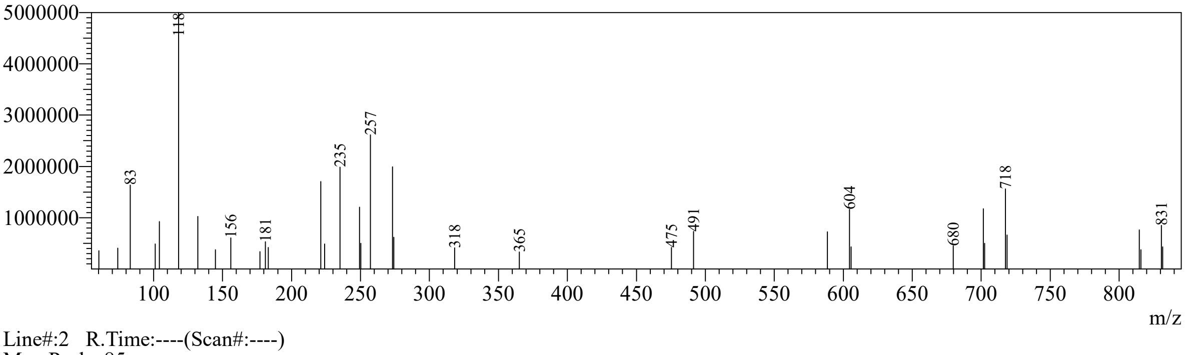

Line#:2 R.Time:----(Scan#:----)
MassPeaks:85
Spectrum Mode:Averaged 0.017-0.930(2-56) Base Peak:387(777232)
BG Mode:None Segment 1 - Event 2

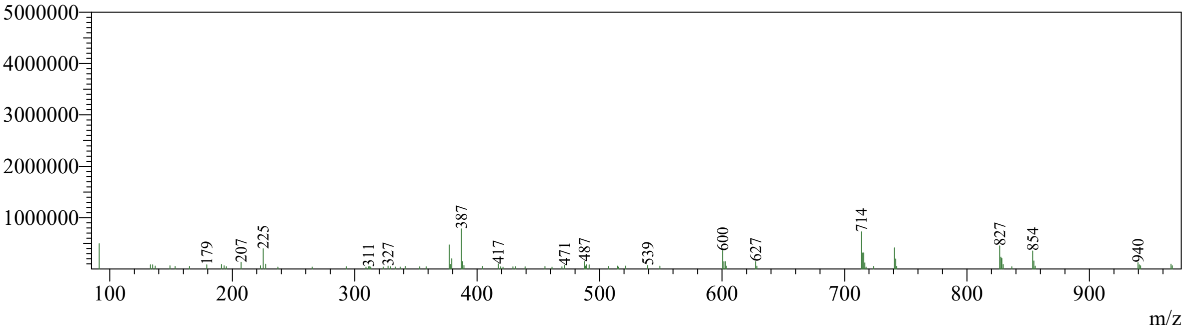

MS Spectrum

Line#:1 R.Time:----(Scan#:----)
MassPeaks:36
Spectrum Mode:Averaged 0.000-0.914(1-55) Base Peak:118(6370435)
BG Mode:None Segment 1 - Event 1

NAWAL ANALYTICAL LABORATORIES HOSUR

#	m/z	Absolute Intensity	Relative Intensity
1	60.25	353782	5.55
2	74.10	408770	6.42
3	83.05	1632509	25.63
4	101.25	485645	7.62
5	104.25	923360	14.49
6	118.20	6370435	100.00
7	132.10	1024069	16.08
8	144.95	374998	5.89
9	155.95	607840	9.54
10	177.15	339400	5.33
11	181.15	528518	8.30
12	183.10	417592	6.56
13	221.20	1705778	26.78
14	224.05	484825	7.61
15	235.15	1987133	31.19
16	249.20	1207332	18.95
17	250.20	502026	7.88
18	257.15	2614346	41.04
19	273.15	1990153	31.24
20	274.25	615206	9.66
21	318.35	412252	6.47
22	365.15	332257	5.22
23	475.40	415405	6.52
24	491.35	730823	11.47
25	588.50	723742	11.36
26	604.50	1162971	18.26
27	605.55	430947	6.76
28	679.65	446881	7.01
29	701.60	1173407	18.42
30	702.55	500515	7.86
31	717.60	1560454	24.50
32	718.60	658636	10.34
33	814.70	760318	11.94
34	815.65	373820	5.87
35	830.60	848908	13.33
36	831.55	433675	6.81

Line#:2 R.Time:----(Scan#:----)
MassPeaks:85
Spectrum Mode:Averaged 0.017-0.930(2-56) Base Peak:387(777232)
BG Mode:None Segment 1 - Event 2

#	m/z	Absolute Intensity	Relative Intensity
1	91.30	495087	63.70
2	133.10	81423	10.48
3	135.05	82197	10.58
4	137.15	58999	7.59
5	149.20	63894	8.22
6	153.15	52191	6.71
7	165.10	51906	6.68
8	179.15	79931	10.28

NAWAL ANALYTICAL LABORATORIES HOSUR

#	m/z	Absolute Intensity	Relative Intensity
9	191.10	89143	11.47
10	193.15	59741	7.69
11	195.15	46476	5.98
12	207.15	128502	16.53
13	223.10	62510	8.04
14	225.15	390267	50.21
15	227.15	92039	11.84
16	237.10	39433	5.07
17	265.20	39583	5.09
18	293.20	43751	5.63
19	309.20	40420	5.20
20	311.25	44199	5.69
21	312.25	50764	6.53
22	313.15	40262	5.18
23	323.15	39982	5.14
24	327.20	55017	7.08
25	329.20	43983	5.66
26	333.15	39214	5.05
27	337.20	39826	5.12
28	341.20	48467	6.24
29	353.20	41642	5.36
30	358.30	43631	5.61
