# Supplementary figures and images for: Integrated Metabolite and Transcriptome Profiling-Mediated Gene Mining of Sida cordifolia Reveals Medicinally Important Genes
Source: Genes (Basel). 2022 Oct 20;13(10):1909. doi: 10.3390/genes13101909 (PMC9602365; doi:10.3390/genes13101909)

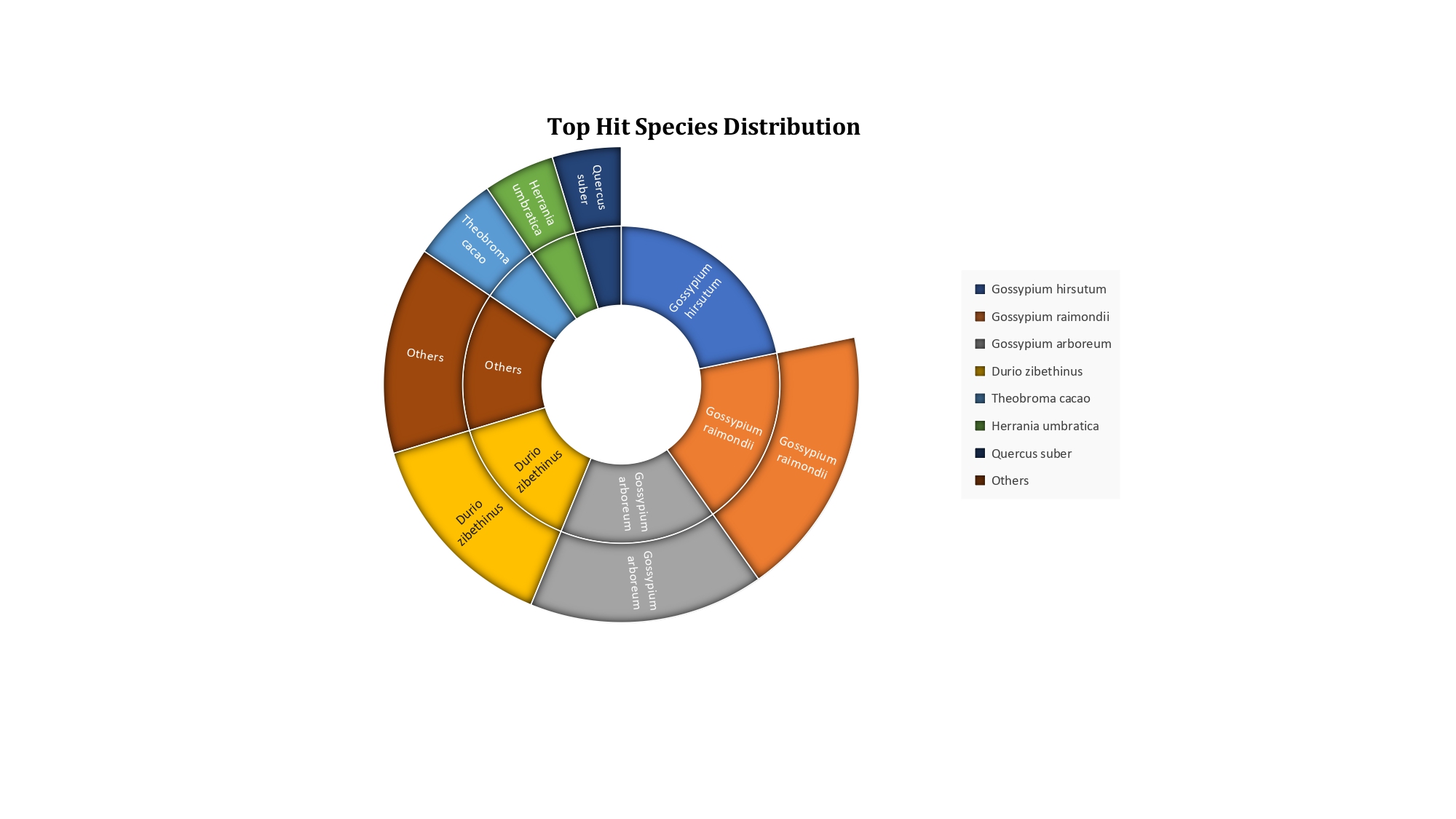

Supplement: Supplementary file 1 [file genes-13-01909-s001.zip › Figure S1.jpg]

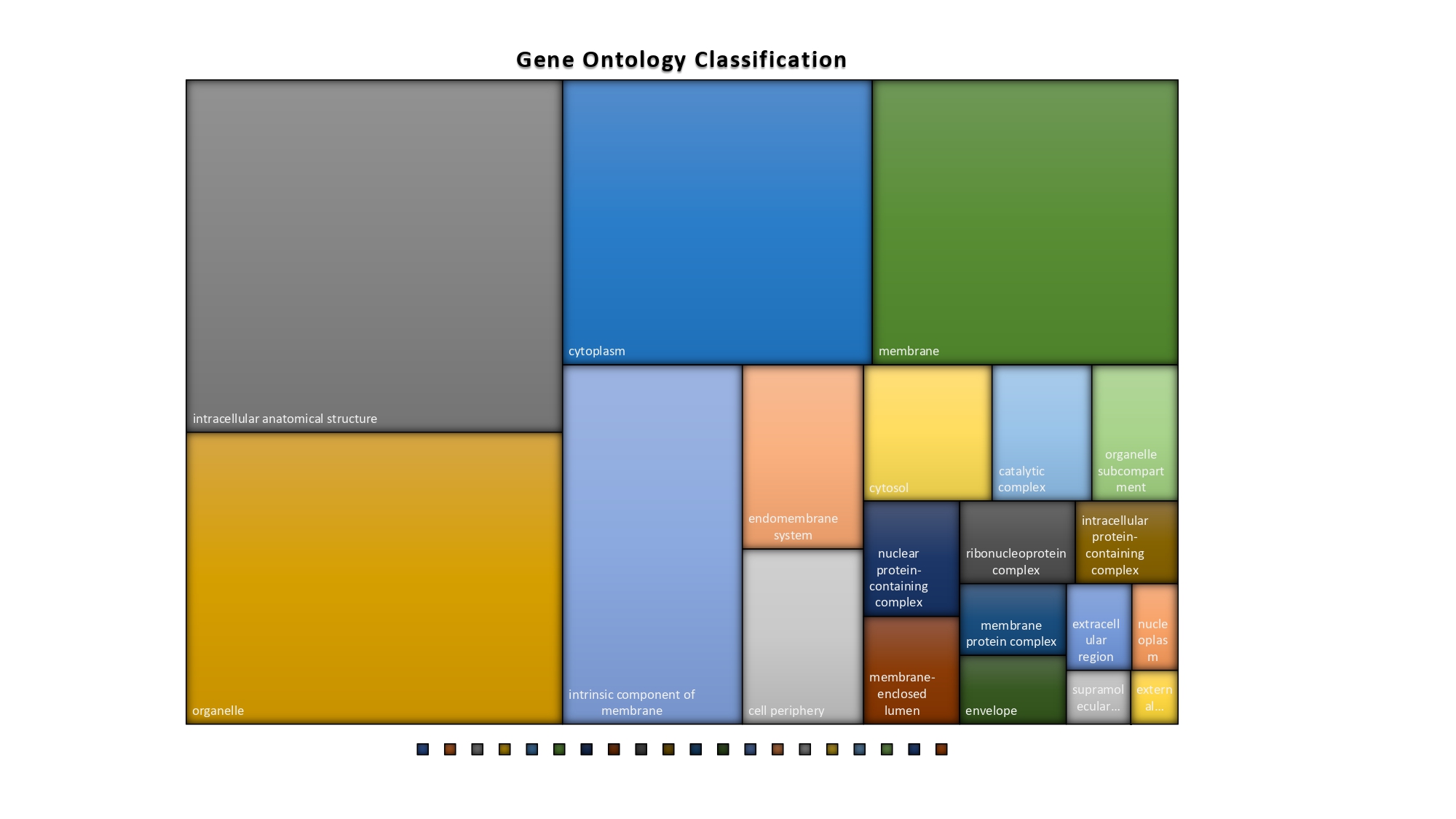

Supplement: Supplementary file 1 [file genes-13-01909-s001.zip › Figure S2.jpg]

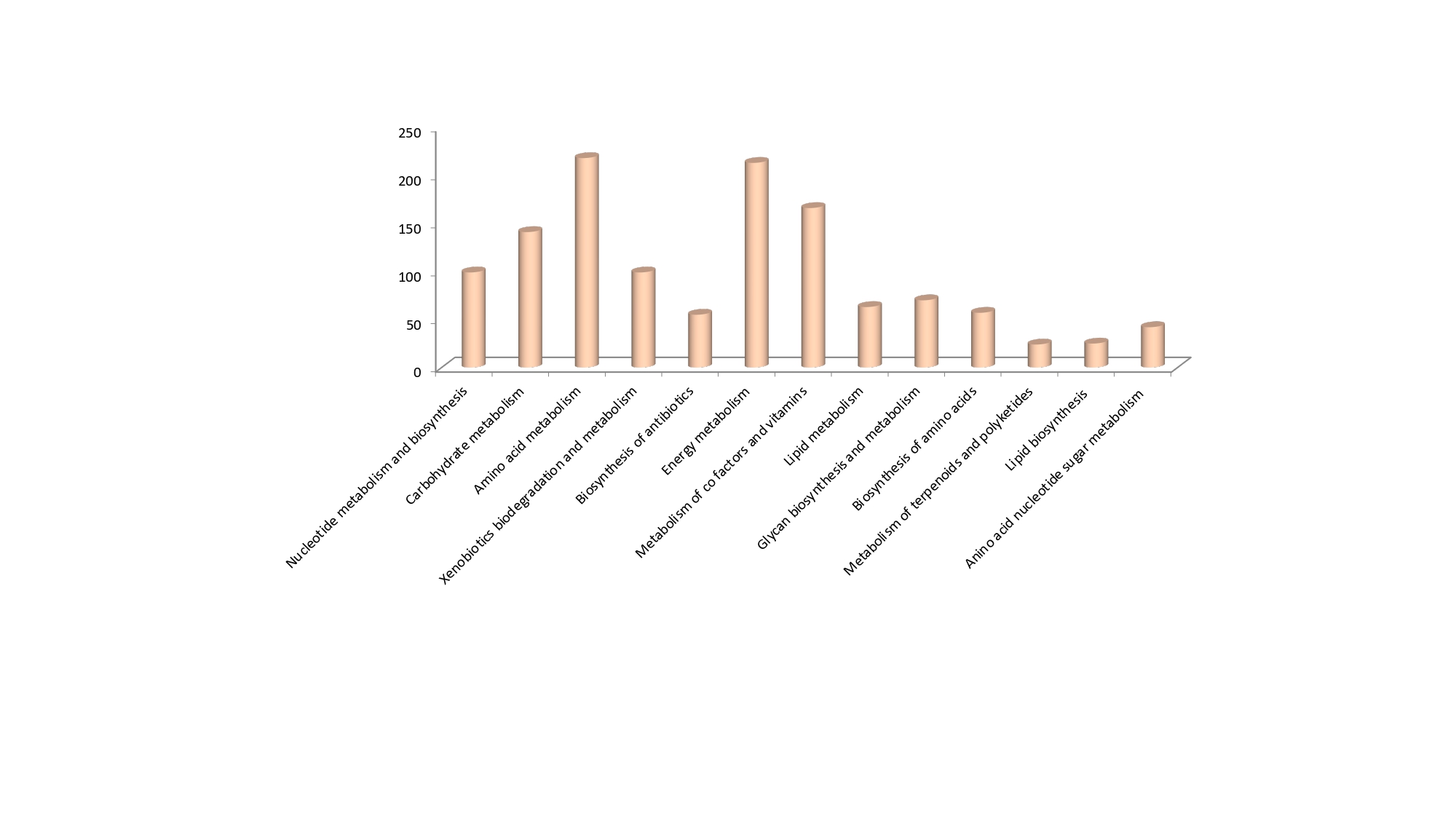

Supplement: Supplementary file 1 [file genes-13-01909-s001.zip › Figure S3.jpg]

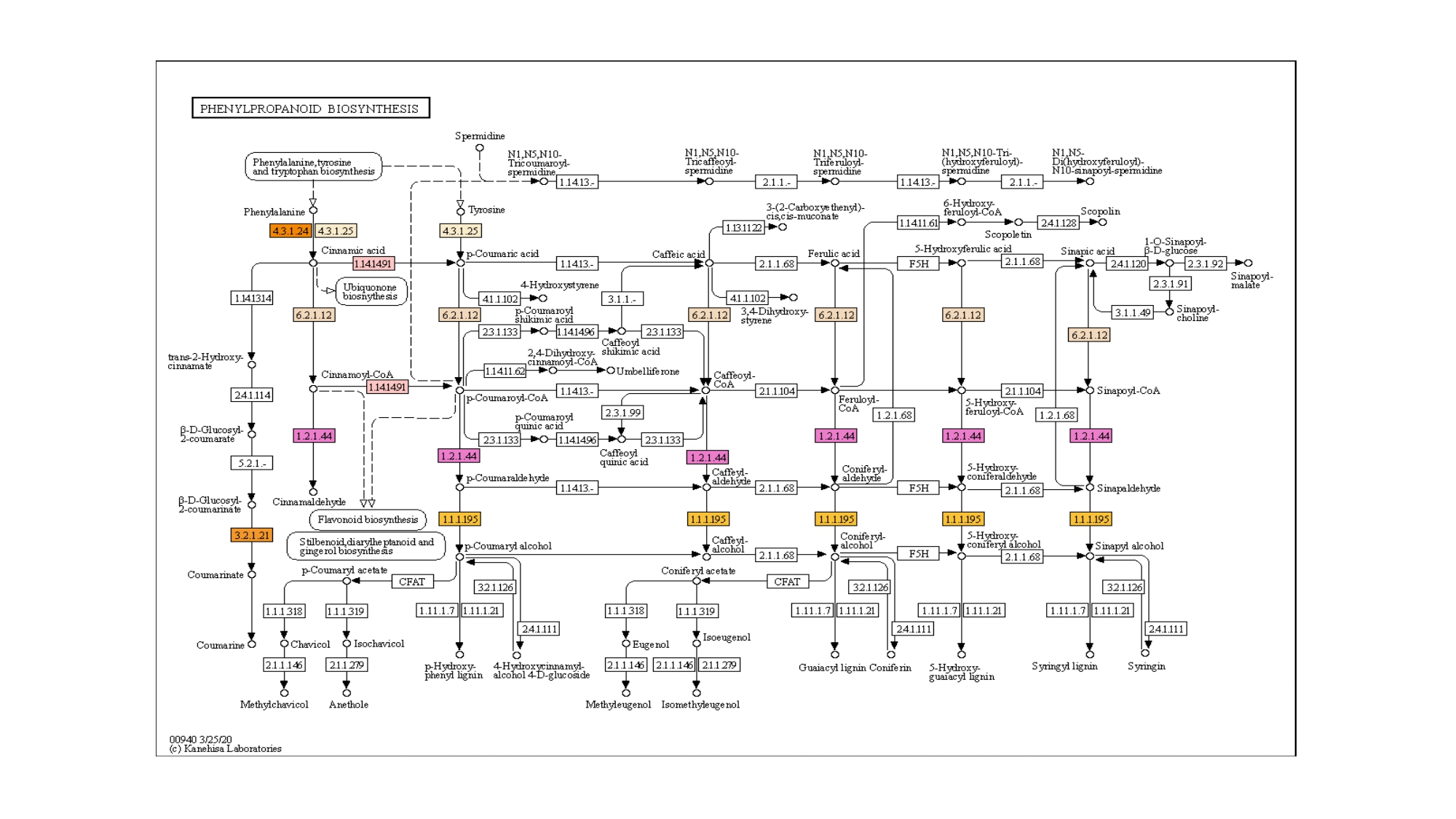

Supplement: Supplementary file 1 [file genes-13-01909-s001.zip › Figure S4.jpg]

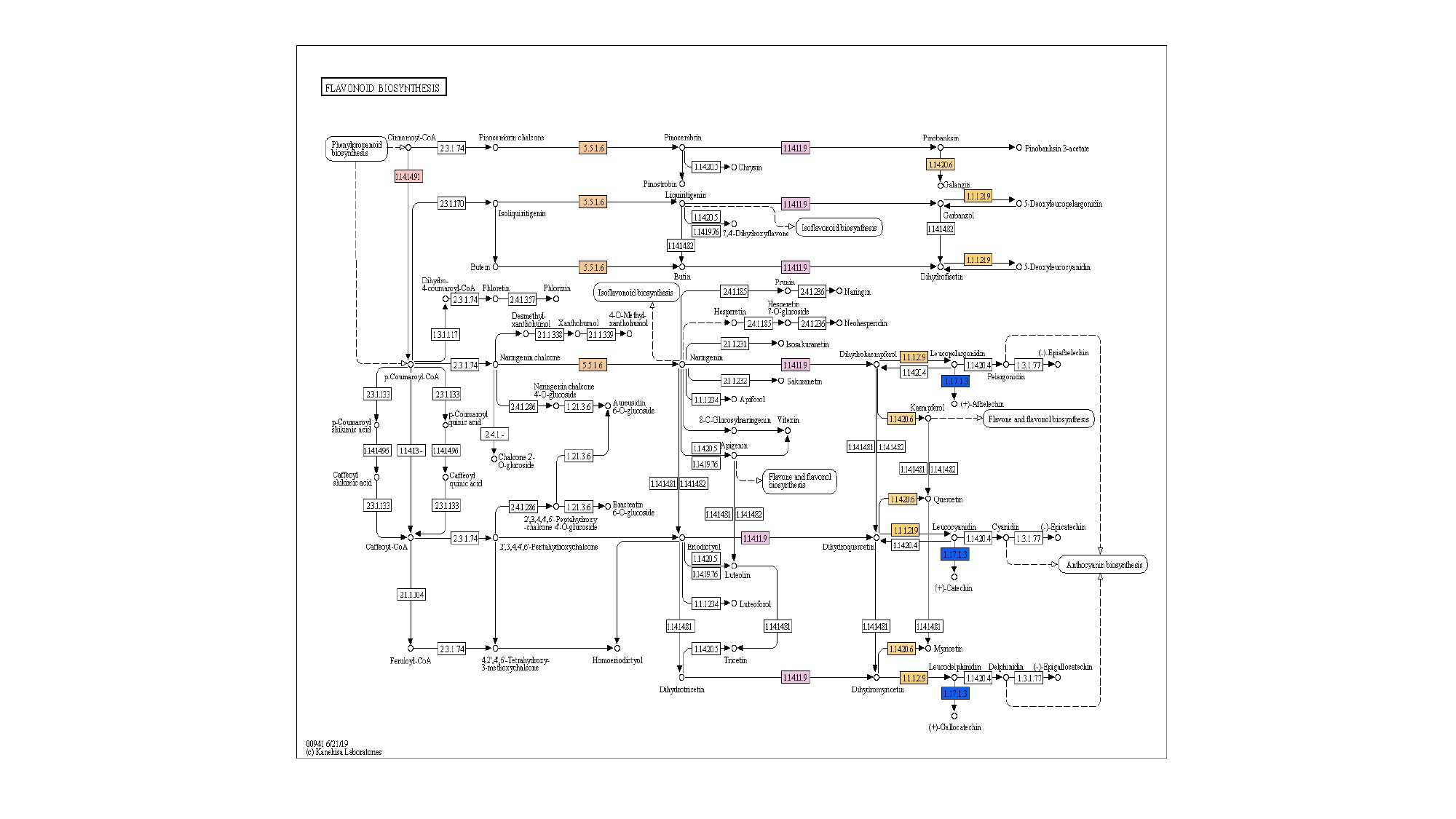

Supplement: Supplementary file 1 [file genes-13-01909-s001.zip › Figure S5.jpg]

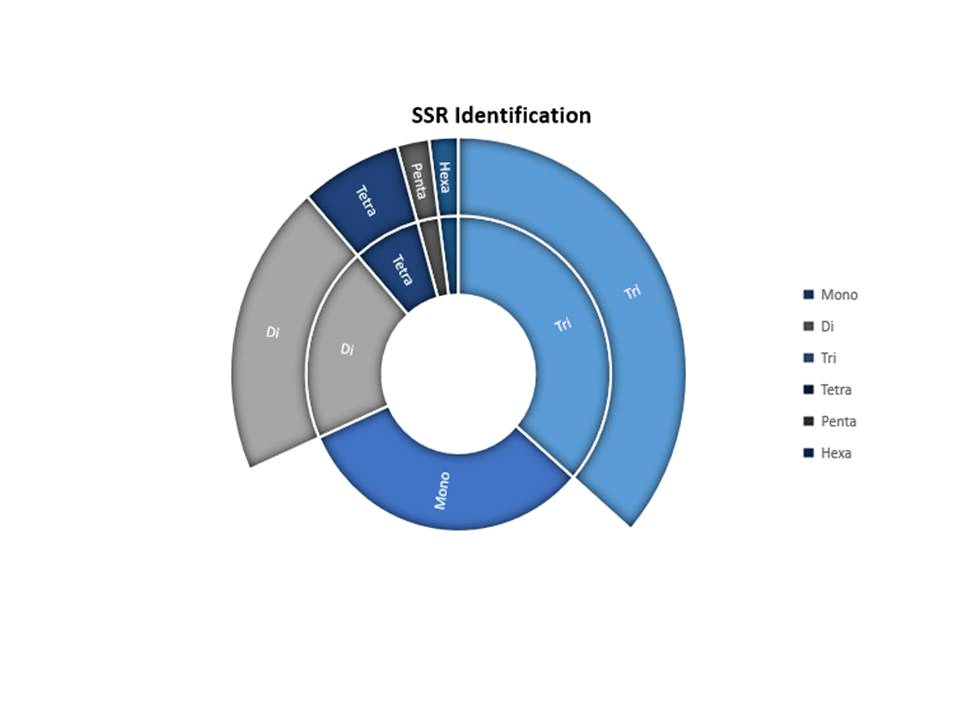

Supplement: Supplementary file 1 [file genes-13-01909-s001.zip › Figure S6.png]
